# Structural investigations on orotate phosphoribosyltransferase from *Mycobacterium tuberculosis*, a key enzyme of the *de novo* pyrimidine biosynthesis

**DOI:** 10.1038/s41598-017-01057-z

**Published:** 2017-04-26

**Authors:** Stefano Donini, Davide M. Ferraris, Riccardo Miggiano, Alberto Massarotti, Menico Rizzi

**Affiliations:** Department of Pharmaceutical Sciences, Università del Piemonte Orientale “A. Avogadro”, Largo Donegani 2, 28100 Novara, Italy

## Abstract

The *Mycobacterium tuberculosis* orotate phosphoribosyltransferase (MtOPRT) catalyses the conversion of α-D-5-phosphoribosyl-1-pyrophosphate (PRPP) and orotate (OA) in pyrophosphate and orotidine 5′-monophosphate (OMP), in presence of Mg^2+^. This enzyme is the only responsible for the synthesis of orotidine 5′-monophosphate, a key precursor in the *de novo* pyrimidine biosynthesis pathway, making MtOPRT an attractive drug target for the development of antitubercular agents. We report the crystal structures of MtOPRT in complex with PRPP (2.25 Å resolution), inorganic phosphate (1.90 Å resolution) and the exogenous compound Fe(III) dicitrate (2.40 Å resolution). The overall structure of the mycobacterial enzyme is highly similar to those described for other OPRTases, with the “flexible loop” assuming a well define conformation and making specific contacts with the Fe(III)-dicitrate complex. The structures here reported add to the knowledge of a potential drug target for tuberculosis, and will provide a useful tool for the structure-based drug design of potent enzyme inhibitors.

## Introduction

Tuberculosis (TB) is a widespread, severe human infectious disease caused by the bacterium *Mycobacterium tuberculosis* (Mtb). Mtb infects 9.6 millions people every year and is a major sanitary concern causing approximately 1.5 million deaths per year. In addition, the rise of multi- and extensively- drug-resistant TB represents a severe and increasing threat for the public health^1–3^. Hence, the identification of new vulnerable drug targets, essential for Mtb survival and having no pre-existing resistance reported, is of high priority for the discovery of novel agents against TB.

Currently available antitubercular drugs target mycobacterial proteins involved in diverse and essential cellular functions such as cell wall synthesis, energy metabolism, protein synthesis and the metabolism of key molecules and cofactors^4–8^. A new class of effective and promising antitubercular molecules (quinolones and fluoroquinolones) targets DNA replication, thus limiting and impairing mycobacterial viability at different stages of the infection cycle^4^. In this context, also the *de novo* and salvage synthesis of purine and pyrimidine nucleotides, the key precursors of DNA and RNA, have been reported as essential for mycobacterial survival both *in vitro* and *in vivo* ^9^, and therefore represents a source of promising targets for the development of new drugs against TB.

In bacteria, the *de novo* synthesis of pyrimidines requires a strict regulation of six enzymes involved in a series of consecutive reactions that ultimately lead to the formation of uridine 5′-monophosphate (UMP), the precursor of all pyrimidine nucleotides^10^. The fifth step in the *de novo* biosynthetic pathway is catalyzed by the enzyme orotate phosphoribosyltransfrase that synthesises orotidine 5′-monophosphate (OMP) and pyrophosphate from α-D-5-phosphoribosyl-1-pyrophosphate (PRPP) and orotate (OA). MtOPRT has been reported to follow a Mono-Iso ordered Bi–Bi kinetic mechanism in which the enzyme undergoes an isomerization of the transitory ternary complex prior to products release^11–15^.

Herein, we report the X-ray crystal structures of MtOPRT (coded by the *rv0382c* gene) in complex with PRPP, inorganic phosphate and an exogenous Fe(III) dicitrate molecule (ferric dicitrate, FDC) at 2.25 Å, 1.90 Å and 2.40 Å resolution, respectively. Since nucleotide biosynthesis is necessary for survival in latent bacilli^12^ and OPRTases are already considered promising targets for the development of new molecules for the treatment of malaria^16^ and toxoplasmosis^17^, these structures add to the knowledge of OPRTases and provide a structural platform for the rational design of MtOPRT inhibitors of potential interest for future development of novel anti-tubercular agents.

## Results

### Protein preparation

Recombinant MtOPRT was purified to homogeneity using a Ni-NTA affinity chromatography followed by size exclusion chromatography yielding about 5 mg of pure and active enzyme per liter of bacterial culture. Consistent with previously reported data^11^, in the final purification step the protein eluted as a single peak with an exclusion volume corresponding to the functional dimeric form of the enzyme.

### Overall Structure of *M. tuberculosis* OPRTase

**Figure 1:**
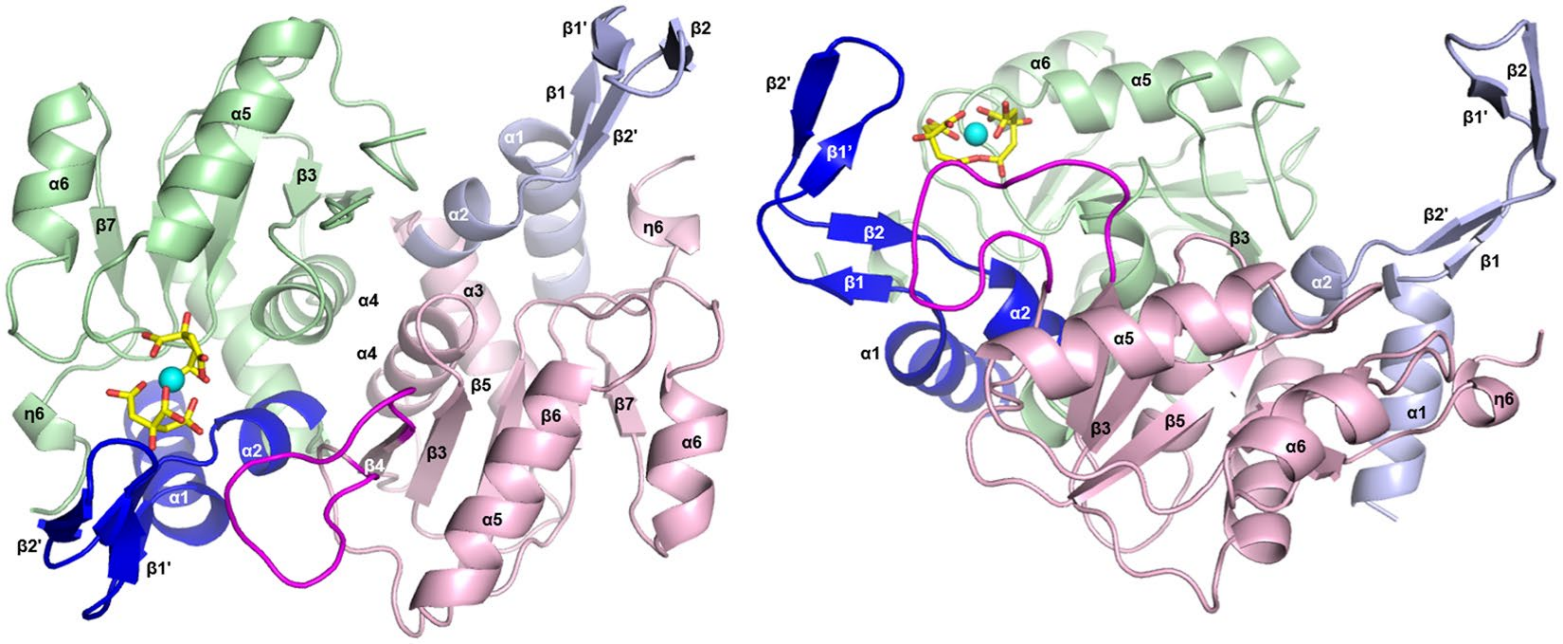
Overall structure of MtOPRT. Cartoon representation of the dimeric MtOPRT structure. Monomers A and B are coloured in green and pink, respectively; hood domains of protomers A and B are coloured respectively in blue and light blue, the ligand FDC is colored in yellow and the Fe(III) is represented as a light blue sphere; flexible loop of chain B is colored in purple.

**Figure 2:**
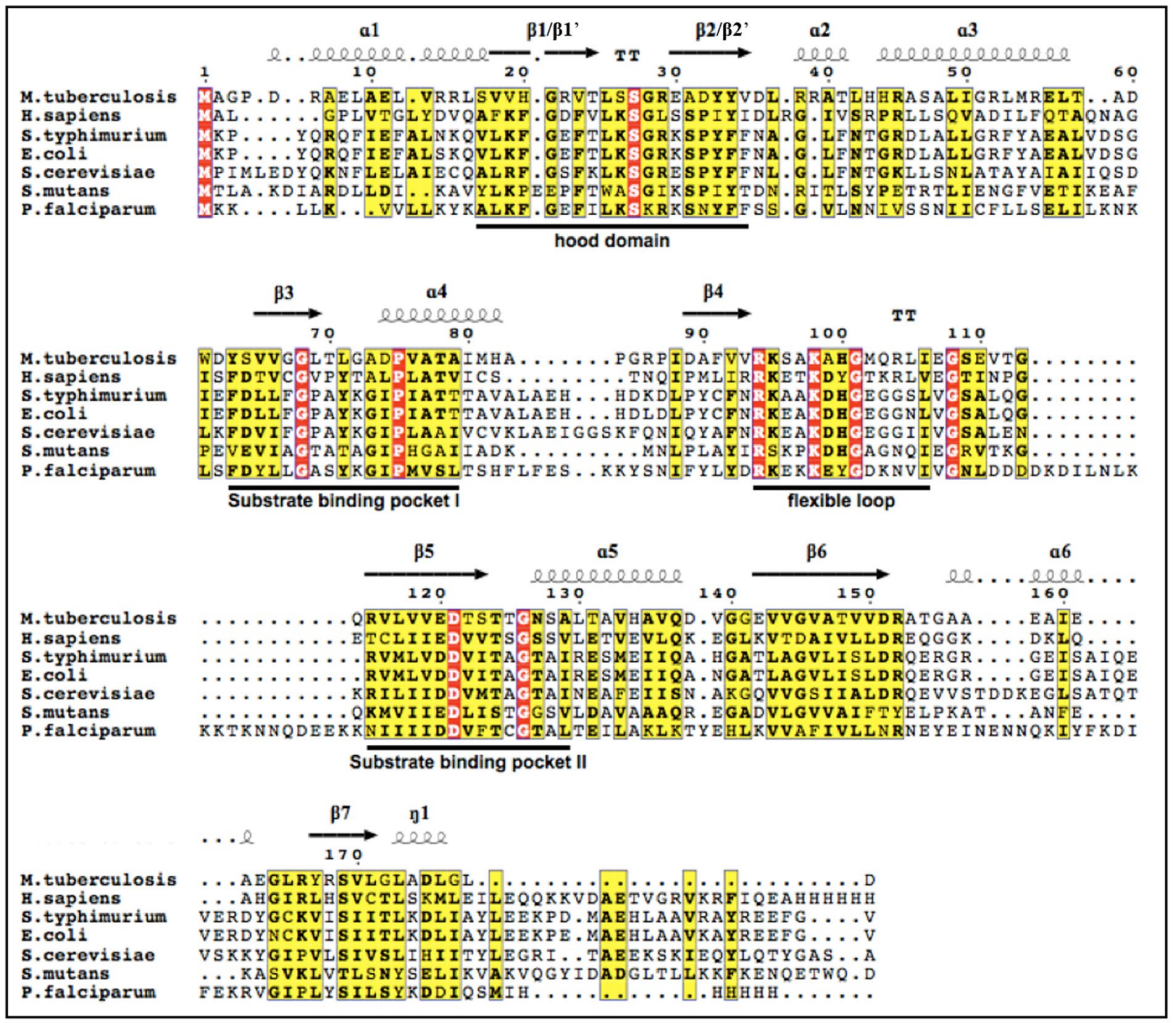
Sequence alignment of various OPRTase. Aligned sequences of OPRTase from *M. tuberculosis* H37Rv, *H. sapiens*, *S. typhimurium*, *E. coli*, *S. cerevisiae*, *S. mutans* and *P. falciparum*. Conserved residues are shown on a red background, whereas chemically similar residues are displayed on a yellow background. Numbering and secondary structure elements assignments, together with the position of hood domain, substrate binding pockets I–II, and the flexible loop, are highlighted for the mycobacterial enzyme.

In all the structures herein described, both monomers (Fig. 1) composing the functional homodimeric unit are related by a 2-fold axis, with a solvent excluded surface between monomers of 1045 Å^2^ (13% of each subunit surface). The interactions across the interface include mainly three regions and involve, for each subunit, helices α2 and α3, the entire helix α4, and the strand β4 (Fig. 2). Both subunits are essentially identical, with a root mean square deviation for all Cα atoms of 0.133 Å; each subunit is composed by seven β-strands (labelled β1–β7), six α-helices (labelled α1–α6), and a 3_10_ helix. A cis-peptide bond formed between Thr70 and Leu71 is present in all the subunits herein described, as already observed for *S. typhimurium* and *S. cerevisiae* OPRTases^18, 19^.

In our structures, several features common to other OPRTases are observed^19–23^. The N-terminal (residues 1–42, blue and light-blue in Fig. 1) is characterized by two antiparallel α-helices (α1–α2) separated by four contiguous β-strands forming two antiparallel β-sheets (residues 18–34). This N-terminal region constitutes the hood domain that undergoes conformational changes instrumental for the formation of the solvent excluded crevice accommodating either the substrate OA or the product OMP. As for the hood domain, a central α/β core (residues 43–169, pink and green for chain A and B, respectively; Fig. 1) represents a common feature for all OPRTases. This domain is composed by five twisted β-strands (β3–β7) surrounded by four α-helices (labelled α3–α6), and it can be divided into two halves. The first half is composed by two parallel β-strands (β3–β4) and two antiparallel α-helices (α3–α4) connected to the second halve by a long loop (residues 94–114). The solvent-exposed mobile region of this loop (residues 96–106) constitutes the so called “flexible loop” that forms the active site of the neighbouring monomer and contributes to ligand binding and catalysis, shielding also the active site from the solvent molecules^19, 21, 24, 25^. Finally, the C-terminal region (residues 172–176) is composed by a single 3_10_ helix that replaces two conserved antiparallel α-helices observed in other OPRTases^19, 21^.

### Binding of PRPP and inorganic phosphate

**Figure 3:**
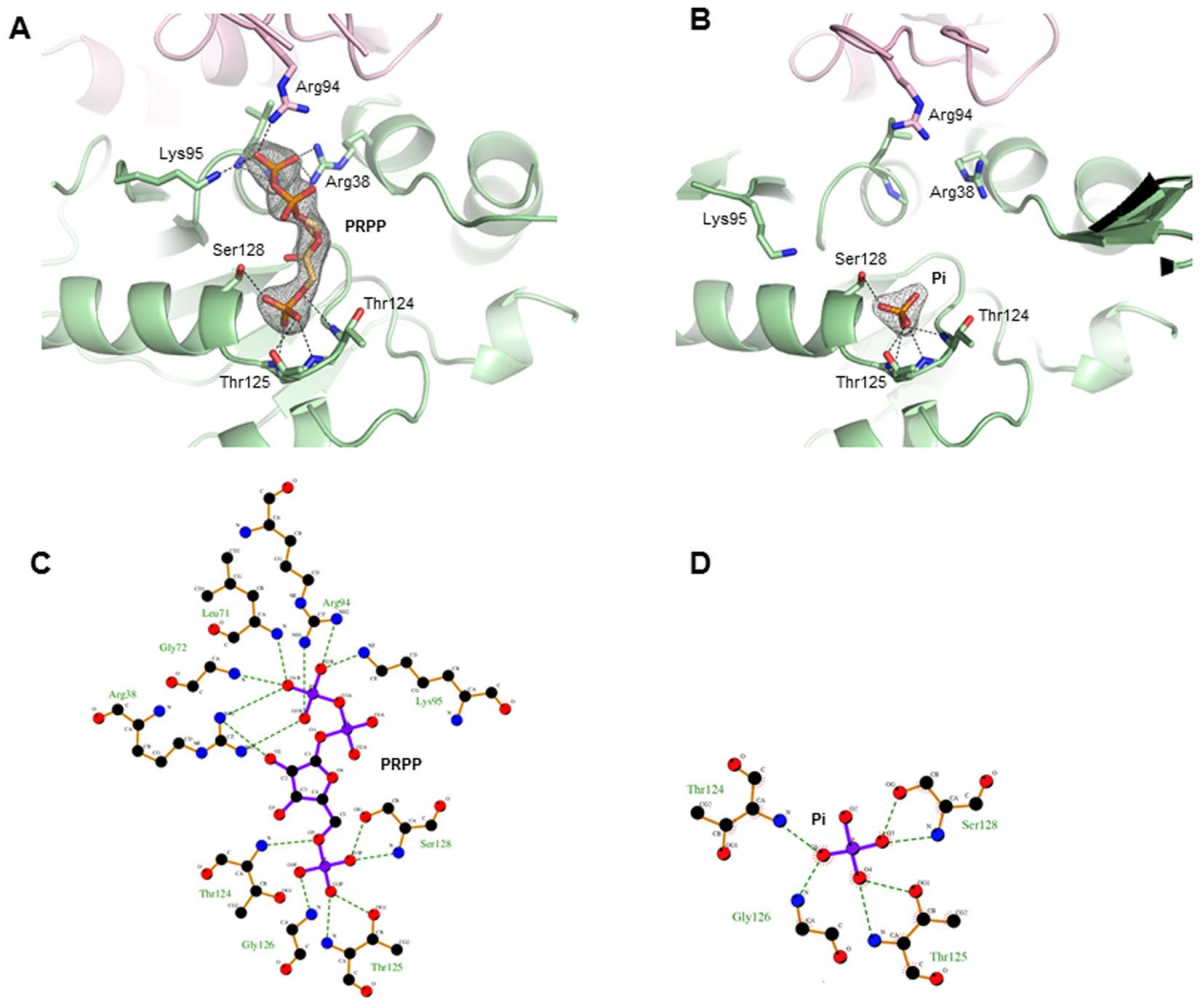
The active site of *M. tuberculosis* OPRTase in complex with PRPP (left hand side; (CC = 0.89) and Pi (right hand side; (CC = 0.97). (**A**) and (**B**)**:** σ_A_-weighted 2Fo-Fc electron density map contoured at 1.2 σ with residues involved in ligand binding depicted as sticks. (**C**) and (**D**)**:** schematic diagrams of the PRPP and Pi binding site. The interactions between ligands and residues within the active site are indicated by dashed black lines.

A PRPP molecule could be unambiguously identified in the active site in both monomers of the binary complex with the substrate crystallized in absence of Mg^2+^ (Fig. 3A). Refinement statistics gave good correlation coefficient score (CC) for the PRPP molecule (CC = 0.89). As observed in the structure of OPRTase from *S. cerevisiae* ^19^ and *S. typhimurium* ^18^, the three oxygens of the PRPP 5-phosphate group interact with the substrate pocket II (also defined as PRPP binding motif, residues 120–128) through a network of hydrogen bonds involving the amide group of the conserved residues Thr124 and Gly126 and the non-conserved residue Thr125. Additional stabilising interactions are provided by the hydroxyl groups of Thr125 and Ser128 (Fig. 3A–C).

An analogous network of hydrogen bonds occurs in the MtOPRT-Pi binary complex (Fig. 3B–D), where the inorganic phosphate occupies the same binding pocket of the 5-phosphate group of PRPP exploiting the same hydrogen-bonds network. Refinement statistics gave good correlation coefficient score for the Pi molecule (CC = 0.97). The ribose hydroxyl groups of the PRPP form hydrogen bonds interactions with Glu120 and Asp121. Glu120 contacts both the substrate O2′ and O3′, while Asp121 interacts only with the O2′.

The PRPP pyrophosphate group occupies the substrate binding pocket I (residues 63–79) establishing a hydrogen bond between its O2β atom and the Nζ atom of Lys95, and a salt bridge between its O3β atom and the guanidino group of Arg38. Finally, the visible and less mobile “flexible loop” moiety from the neighbour monomer participates in substrate binding through several basic residues, with Arg94 directly interacting with the pyrophosphate O2β and O3β atom, as already observed in other OPRTases^19, 21, 24^.

### Binding of Fe(III) dicitrate

The inspection of the catalytic sites of the protein crystallized in absence of ligands revealed a bulky residual density located in the substrate-binding pocket I of one monomer of each of the two functional dimers present in the asymmetric unit. All efforts to unambiguously assigned such residual electron density to any of the substrates or products of the catalytic reaction proved unsuccessful. We interpreted the unexplained electron density as Fe(III) dicitrate (FDC) based on the following assumptions: 1) ferric chloride and sodium citrate were both present in the crystallization buffer; 2) citrate is a well-known iron chelator^26^; 3) speciation diagrams of the citrate-Fe(III) complex in aqueous conditions showed that the mononuclear complex is compatible with the ligand-free MtOPRT pH crystallisation condition^26^ and it is the most physiologically-relevant species of the ferric-citrate complexes^27^.

FDC was manually fitted in the 2Fo-Fc map. Several manual fitting and refinement cycles gave satisfactory results in terms of R factors and geometry statistics. To further validate our fitting model, we fed the automatic-fitting program LigandFit of the PHENIX software suite with the refined, ligand-free protein and FDC coordinates and geometry restrains as input files. Automatic fitting statistics gave satisfactory correlation coefficient score (CC = 0.82) giving a FDC conformation that matched the one modelled manually. Hence, both manual and *in silico* ligand fitting approaches gave convincing, identical results. Moreover, the automatic fitting program modelled the same FDC conformation in both binding sites, further supporting the consistency and reliability of the ligand fitting.

**Figure 4:**
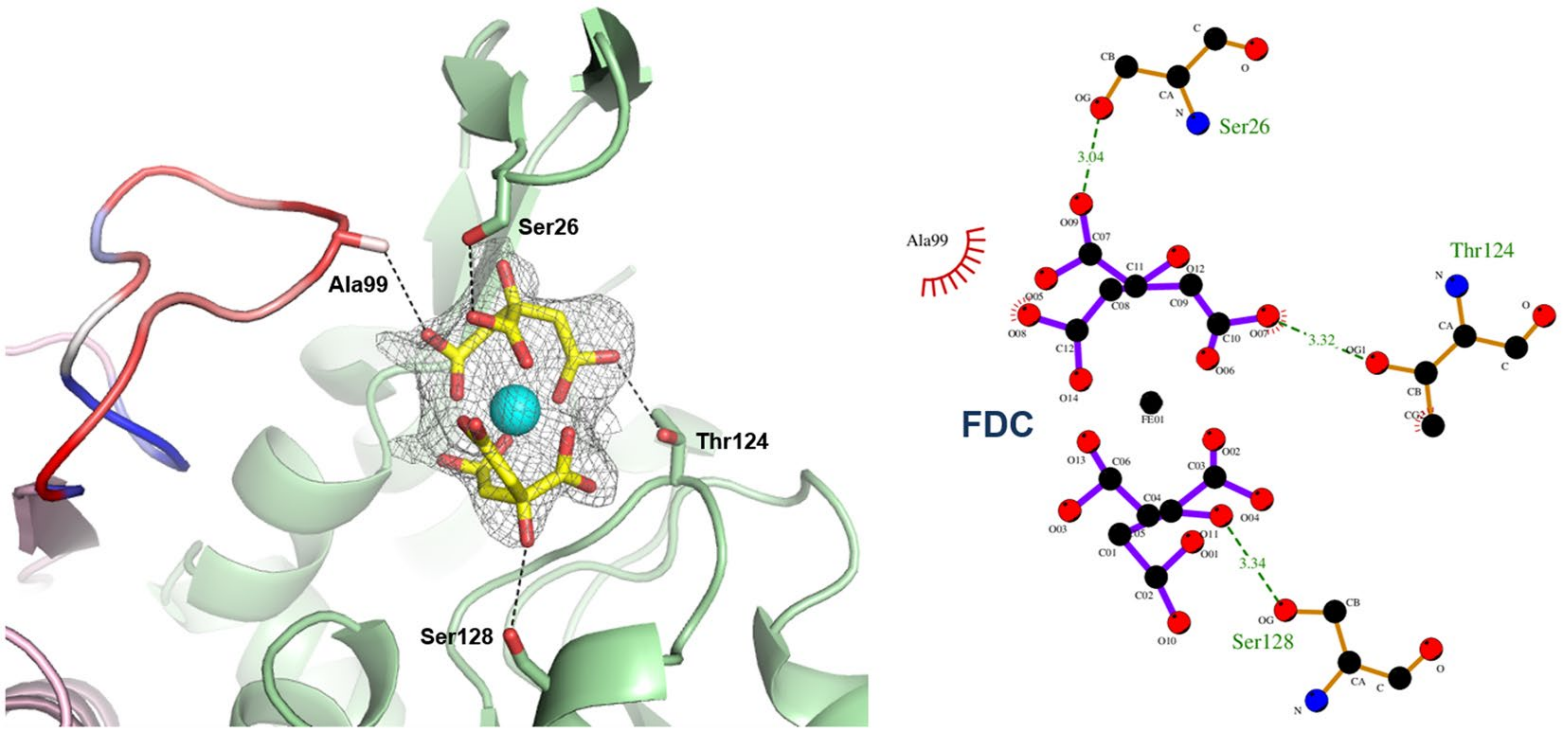
*M. tuberculosis* OPRTase active site in complex with FDC. Left: calculated unbiased omit map. The flexible loop is coloured according to B-factors values in a blue-white-red scale (from low to high values). Interactions between ligand and active site residues are shown as dashed black lines. Right: interaction diagram showing the residues involved in FDC binding. Distances are expressed in Ångstroms.

To rule out the presence of any model bias, we calculated the omit map using the “Map” tool of the PHENIX program suite^28^ based on the refined, ligand-free protein structure. The resulting omit map is fully compatible with the presence of FDC in the modelled conformation (Fig. 4).

As multinuclear iron-citrate forms can co-exist in solution^26^, we also attempted manual and automatic ligand fitting of a dinuclear iron(III) dicitrate species. However, both approaches gave unsatisfactory results, poor fitting scores (CC < 0.7) and refinement statistics, prompting us to conclude that a mononuclear iron dicitrate is present in the MtOPRT active site.

One of FDC iron-binding citrate molecule positions itself in the OMP (or OA) binding site^19, 29^, where the second citrate molecule extends itself towards the hood domain. FDC is held in place by hydrogen bonds involving the non-conserved Ala99 (3.5 Å, long range H-bond). These interactions contribute to the stabilisation of the “flexible loop” of the neighbouring chain (Fig. 4). Moreover, FDC O7(2) interacts with the amidic N- and O-atoms of Thr124 (3.1 Å and 3.2 Å, respectively), while the Oγ of the conserved Ser26 forms hydrogen bond with O1(2) of FDC (3.3 Å). Noteworthy, in our MtOPRT structure, the conserved Ser26 is shown to exclusively interact with the FDC molecule, and does not participate in either OA, OMP or PRPP binding in MtOPRT orthologs^19, 22, 29^.

### Conformational changes upon ligand binding

**Figure 5:**
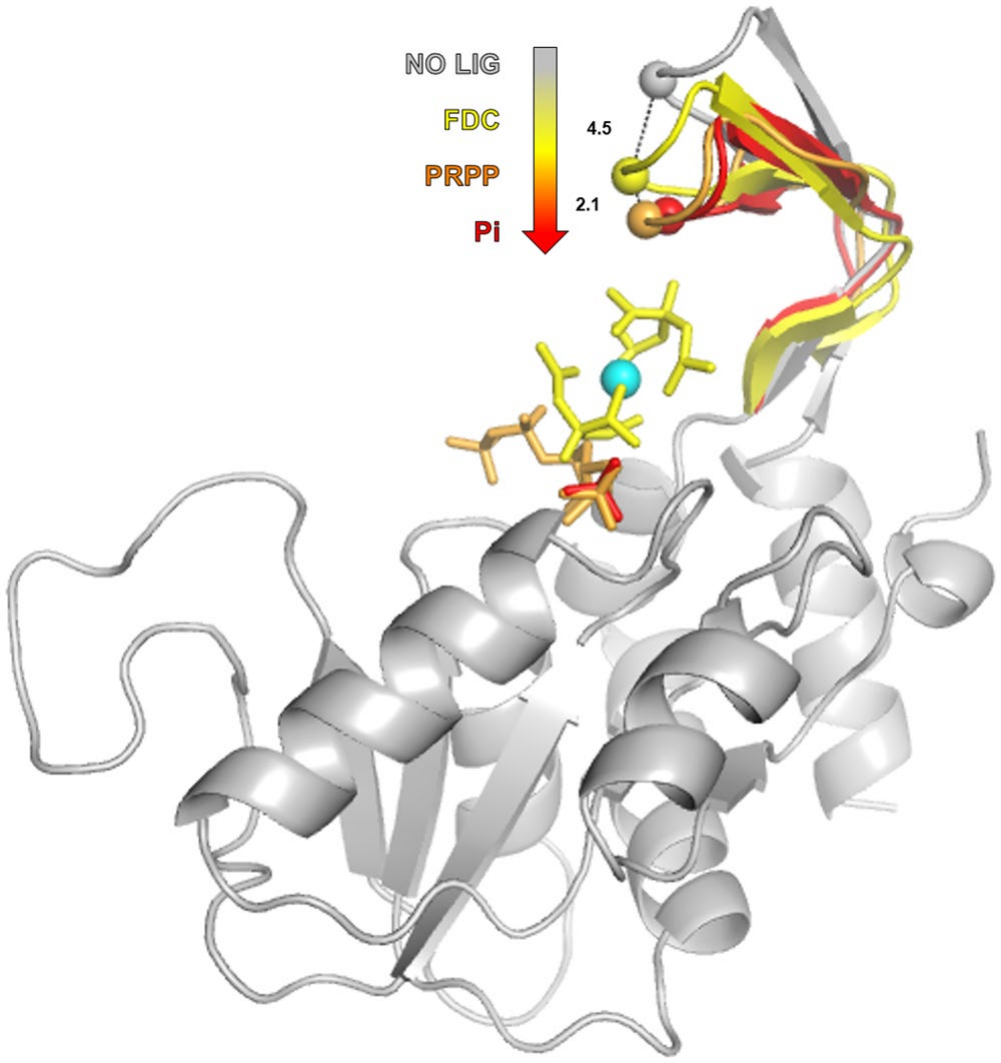
Conformational changes in the OPRTase. Superposition of the monomeric structure of ligand-free MtOPRTase monomer (grey) and in complex with FDC (yellow), PRPP (orange) and Pi (red). The arrow highlights the movement of the hood domain according to the ligand present in the active site. Ser26 Cα’s are shown as spheres according to ligand and structure colours; distances between Ser26 Cα’s are expressed in Ångstroms.

During the catalytic reaction, OPRTases undergo large conformational changes switching from an open to a closed state. In this transition, the hood domain and the flexible loop show the most prominent structural change^19, 22, 24^. All the structures of MtOPRT reported here show a highly similar overall conformation, with a r.m.s.d. of all Cα pairs of about 0.113 Å. However, a significant change can be observed for the hood domain that moves toward the active site after ligand binding generating the solvent-excluded catalytic core described in other OPRTases^19, 22^ (Fig. 5). Interestingly, the extent of the movement varies depending on the different bound ligand: PRPP and Pi cause a bend of approximately 6 Å, and FDC of about 4 Å (based on the position of the Cα of Ser26).

In order to analyse the conformational changes of the hood domain in relation to the bound ligands, we performed molecular dynamics (MD) simulations of the MtOPRT structures focusing our attention on the conformational changes of the hood domain upon ligand binding. As detailed in Supplementary Materials, MD simulations showed that, while the overall structures of the ligand-bound MtOPRT proteins remained essentially identical during the whole simulations, the hood domain exhibited a high flexibility eventually adopting a preferred conformation not observed in the crystal structures and irrespective of the ligand present in the active site.

### Thermal shift analysis of MtOPRT in presence of Fe(III) and citrate ions

Our structural model proposes that the Fe(III)-citrate molecule binds to the MtOPRT catalytic site, hence acting as an inhibitor. To substantiate our model, we performed inhibitory experiments measuring MtOPRT catalytic activity and following addition of increasing concentration of Fe(III) in a citrate-buffered solution. However, activity slopes could not be reliably measured due to the low enzymatic activity of MtOPRT in citrate buffer (pH = 6.1) and to the overlapping absorption spectra of Fe(III) and OA at a wavelength of 295 nm. For these reasons, the inhibition data were deemed not sufficiently robust to allow a reliable determination of the IC50.

**Figure 6:**
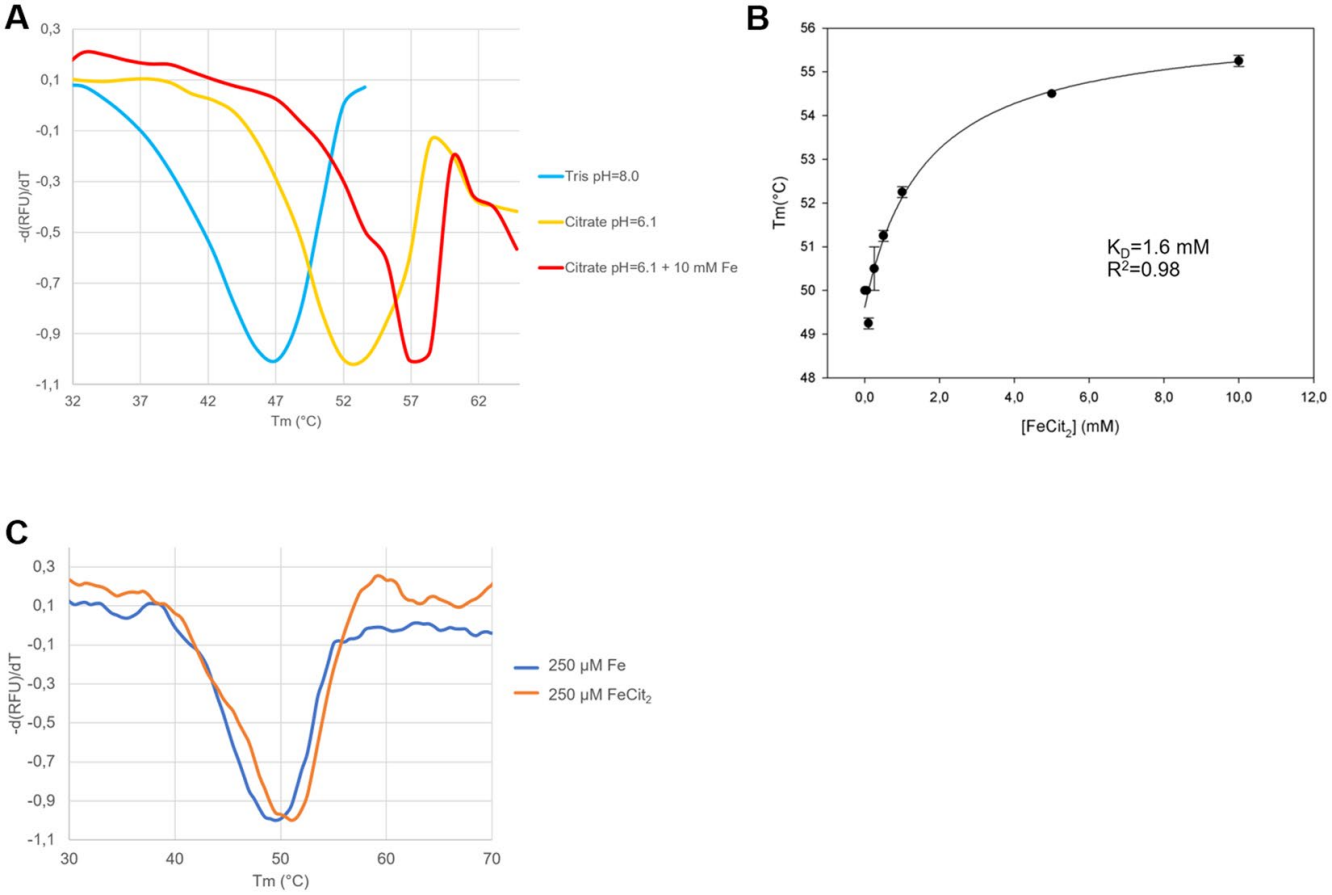
MtOPRT thermal shift experiments shown as derivative data. (**A**) Stabilizing effect of citrate buffer pH = 6,8 and 10 mM Fe(III) on MtOPRT Tm. (**B**) Affinity constant of MtOPRT toward FDC obtained by adding a stoichiometric 1:2 Fe(III)-citrate mix to the protein solution. (**C**) Stabilising effect of Fe(III) alone (blue curve) and of the 1:2 Fe(III)-citrate mix (orange curve) over MtOPRT Tm (ΔTm = 4,5 °C).

In order to circumvent these experimental limitations, we analyzed and quantify both the separate and combined effect of Fe(III) and citrate molecules on protein stability by performing thermal shift assays on MtOPRT and measuring the protein melting temperatures (Tm). These experiments showed that citrate buffer (pH = 6.1) had a stabilizing effect on MtOPRT (Tm = 52 °C) compared to Tris buffer at pH = 8 (Tm = 45,6 °C; ΔTm = 6.4 °C). Moreover, the addition of Fe(III) to the citrate-buffered protein solution further stabilized MtOPRT, causing a shift of Tm from 52.0 °C to 56.8 °C (Fig. 6A).

With the aim of quantifying the affinity of the FDC complex towards MtOPRT, we prepared a solution containing Fe(III) chloride and sodium citrate solutions in a 1:2 stoichimetric ratio, and then measured the Tm shifts following the incremental addition of the stoichiometric Fe(III)-citrate mix to the protein solution. Experimental analysis produced measurable shifts of the Tm, allowing the calculation of the affinity constant (K_D_ = 1.6 mM; Fig. 6B).

Finally, the contribution of Fe(III) alone over protein stability was analyzed by thermal shift experiments by addition, to the protein solution, of the Fe(III) ion in absence of citrate. As shown in Fig. 6C, the Fe(III) alone did not have stabilizing effect at 250 µM (Tm = 49,5 °C), while the stoichiometric Fe(III)-citrate 1:2 mix at a concentration of 250 µM increased the Tm to 54 °C (ΔTm = 4,5 °C).

Hence, coherently to our model, the best stabilizing effect was obtained when Fe(III) and citrate were present in solution in a 1:2 ratio, thus supporting our interaction model and suggesting an exclusive stabilizing effect for the Fe(III)-citrate mix.

## Discussion

We report here the first crystal structures of OPRTase from *Mycobacterium tuberculosis* (PyrE, or Rv0382c) in complex with either PRPP, inorganic phosphate or the exogenous molecule Fe(III) dicitrate. MtOPRT structure is highly similar to that observed for other OPRTases, with the only significant difference in the C-terminal region consisting in the Mtb enzyme of a single 3_10_ helix that replaces the two antiparallel α-helices present in the other orthologs.

The analysis of the enzyme structures in complex with PRPP and Pi lead to the identification of the molecular determinants responsible for ligand recognition and binding. Moreover, the whole flexible loop could be fully traced, allowing to define more precisely its structural role. In particular, we observe that Lys98, belonging to the flexible loop, and whose key role in catalysis has been proved for *S. thyphimurium* OPRTase by site directed mutagenesis^18^﻿, is involved in PRPP binding, as already observed for *S. cerevisiae* ^19^ and *S. thyphimurium* ^25^.

In all our structures the hood domain adopts distinct conformations, confirming, even for the mycobacterial enzyme, its role in the formation of the solvent-excluded active site. MD simulations analysis (see Supplementary Materials) evidenced the high flexibility of the hood domain, while the overall structure remained essentially identical during the whole simulation. In addition, MD analysis showed that the hood domain eventually adopts a preferred conformation irrespective of the ligand present in the active site (see Supplementary Materials Figure S3 relative to the 50 ns MD simulation and accompanying video). This contrasts with what observed in the ligand-bound MtOPRT crystal structures, where the hood domain assumes well-defined, distinct conformations (different from the one eventually adopted in the MD simulations). However, this apparent discrepancy can be rationalized by considering that the MD simulations model the protein structures according to a physiological environment, while the crystal structures, for their proper nature, refer to environmental conditions that are distant from a physiological setting. Hence, the three distinct conformations adopted by the hood domain in a non-physiological environment (such as the crystal packing and the crystallization conditions) could be considered as three “snapshots” representative of the multiple conformations of the hood domain (visible at around 20 ns in the MD simulation, see Supplementary Materials Figure S3) that have been trapped during crystallization and captured by the crystal structures.

### Presence of Fe(III) dicitrate complex in the catalytic site: rationale and implications

The serendipitous binding of ferric dicitrate species in the protein catalytic core gave rise to speculations over its presence and its role in catalysis.

Iron and citrate are ubiquitous molecules in nature and play essential roles in the organisms’ biology^30, 31^. Citrate is a well-known iron chelator and its chemistry and chelating properties, due to their complexity, are still a matter of investigations^27^.

The composition of ferric citrate solutions is complex since multinuclear metal species can form^32^. Recent studies indicate that the mononuclear iron-citrate complexes are the most abundant at physiological pH values and the most important in natural processes^33^. In particular, the predominant ferric-citrate complex species at physiological pH values are represented by monoiron dicitrate complex and multinuclear species of low nuclearity^26^. Given the predominant nature of the mononuclear dicitrate species at physiological conditions and the presence of iron(III) and citrate species in the crystallisation solution of ligand-free MtOPRT, we manually fitted the FDC molecule in the electron density with satisfactory results both in terms of ligand geometry and refinement statistics. Manual ligand fitting was confirmed by automatic ligand fitting procedures, giving identical position of the ligand molecules in the electron density. More convincingly, the modelled FDC molecules fitted the unbiased omit map of the ligand-free MtOPRT, with the protruding hydroxy groups matching the map peripheral lobes. (Fig. 4).

Fitting of dinuclear iron species proved worse in terms of ligand geometries, fitting scores and refinement statistics. For these reasons, manual and *in silico* modelling of multi-iron species with number of Fe(III) >2 were not attempted. Therefore, we conclude that, based on the rationale given by the experimental conditions and on the manual and automatic ligand fitting and refinement statistics, the bound species is monoiron dicitrate. However, we cannot fully rule out the presence in the crystal of other co-existing multinuclear, multi-citrate iron complexes with various stoichiometries compatible with the crystallisation conditions. Although unlikely, the heterogeneity of the Fe(III)-citrate complexes in the crystal could partially explain the presence of residual density in the Fo-Fc map still present in the refined Fe(III) dicitrate complex. The residual density could be fully explained by fitting water molecules in the electron density mimicking the hydration shell of the FDC complex. However, due to the non-unambiguous placement of the water molecules in the bulk electron density, these were omitted from the final model and refinement. Moreover, in order to further support the structural data, we performed thermal shift experiments that showed a stabilizing effect of FDC, not matched by either Fe(III) or citrate alone, with a K_D_ of 1.6 mM, well below the concentration of Fe(III)-citrate present in the crystallization buffer.

The presence of Fe(III)-dicitrate species in the catalytic site has intriguing implications over the catalytical modulation of enzyme activity. Additionally, the residue Ala99, non-conserved in the OPRTase family and present only in the mycobacterial protein, stabilizes the flexible loop through an hydrophobic and specific interaction with Fe(III)-citrate. This unique structural feature could be exploited for the development of a new class of potent and selective MtOPRT inhibitors.

Organometallic compounds represent a new class of drugs that holds promise for the development of novel antibacterial agents with limited antimicrobial resistance^34^. The study of new organometallic molecular scaffolds for anti-mycobacterial drug development could represent a new research area for the design of new, active molecules^35^.

In the context of TB, where antimicrobial resistance can lead to the onset of multidrug- and extensively drug-resistant Mtb strains, the study and development of new organometallic inhibitory molecules offers unprecedented opportunities for the development of new antitubercular drugs.

In conclusion, the here described protein models represent the first structural report of OPRTase from *Mycobacterium tuberculosis* that could be exploited for the engineering of new compounds active against MtOPRT catalysis and for the development of new antitubercular drugs.

## Methods

### Expression and Purification of MtOPRT

The rv0382c gene encoding for MtOPRT was PCR amplified from *Mycobacterium tuberculosis* H37Rv genomic DNA (Table 1) and cloned into a pCold™ I DNA vector (Takara Bio Inc.) by conventional methods leading to the synthesis of an N-terminal 6xHis-tagged protein. The constructed plasmid was transformed into *E. coli* BL21(DE3) cell pGro7 (Takara Bio Inc.) for protein expression. MtOPRT was expressed in presence of human groES-groEL in a 2XTY medium supplemented with ampicillin (50 µg/ml), chloramphenicol (34 µg/ml), and arabinose (1 mg/ml). Bacteria were precultured overnight in 2XTY medium, re-diluted in the same medium, and incubated at 37 °C. The absorbance was constantly monitored until it reached an OD_600nm_ = 0.6. The temperature was then lowered to 15 °C and protein expression was induced by the addition of 0.5 mM isopropyl 1-thio-β-D-galactopyranoside, and culture was incubated for 20 hours. Cells were then harvested by centrifugation, resuspended in buffer A (Tris-HCl 50 mM pH 8.0) supplemented with protease inhibitor cocktail and Benzonase nuclease (Sigma-Aldrich), and lysed by sonication on ice. The lysate was then clarified by centrifugation, and the soluble fraction was loaded onto a Ni-NTA column equilibrated with buffer A. After a washing step with five column volumes of buffer A supplemented with 40 mM imidazole, the enzyme was eluted with the same buffer supplemented with 250 mM imidazole, and fractions containing MtOPRT were pooled and concentrated. The concentrated pool was then loaded on a gel filtration high resolution column (HiPrep 16/60 Sephacryl 200) equilibrated with buffer A. The protein eluted in a single peak and the corresponding fractions were collected and concentrated to 5.4 mg/ml. All the purification steps described above were performed at 4 °C and the respective quantitative details are reported in Table 2. Protein concentration was determined using the Bradford protein assay^36^, and sample purity was assessed by 10% SDS-PAGE.

**Table 1:** Primers used in this study.

Primers	Primer sequence (5′-3′)
MtOPRT-Fw	gaacatatggccggacctgaccgcgcagagttggc (underlined NdeI site)
MtOPRT-Re	gcggatccctaatccagccccagatcggccaggcc (underlined BamHI site)

**Table 2:** Protein purification procedure with yield for each step (1 L of *E. coli* culture).

Purification Step	Total Protein (mg)	Total Activity (U)	Specific Activity (U/mg)	Fold Purification	Yield (%)
Crude Lysate	194	388	2	1	100
Ni-NTA^+^	14.5	188.5	13	6.5	48.6
S-200	6.25	93.75	15	7.5	24
Final MtOPRT	5.25	89.25	17	8.5	23

### Enzyme Activity Assay

Orotate phosphoribosyltransferase activity was assayed as previously described^11^. The reaction mixture contained 50 mM Tris HCl buffer pH 8.0, 20 mM Mg_2_Cl, 10 mM orotic acid and 10 mM PRPP (Sigma Aldrich). The reaction was started by the addition of MtOPRT after 1 minute of incubation and followed by the decrease in absorbance at 295 nm caused by the conversion of orotic acid (OA, ε_295_ = 3950 M^−1^ cm^−1^) in orotate mononucleotide phosphate (OMP). One unit was defined as the enzyme required to convert 1 µmol of OA in OMP per minute and the specific activity was expressed as U mg^−1^ of enzyme. The assay was performed in a quartz cuvette using a Varian Cary 50-BiO UV-visible spectrophotometer equipped with a temperature controlled cuvette holder.

### Thermal Shift Assay

The reaction mix used in the thermal shift assay consisted of the MtOPRT protein diluted to a final concentration of 0.5 mg/mL and with the fluorescence probe SYPRO Orange (Sigma–Aldrich) at 1/4000 dilution. The final reaction volume was 20 µl. Fluorescence was recorded in a 48-well plate using the FAM channel of a MiniOpticonTM Real-Time PCR Detection System (Bio-Rad). Data were harvested and analyzed using CFX ManagerTM Software (Bio-Rad) and SigmaPlot (Systat Software, San Jose, CA, www.sigmaplot.com).

### Multiple sequence alignment

Aminoacidic sequences of OPRTases from M. tuberculosis H37Rv, *Homo sapiens, Escherichia coli, Salmonella typhimurium, Saccharomyces cerevisiae, Streptococcus mutans* and *Plasmodium falciparum* were aligned using T-Coffee Expresso online tool^37, 38^ and displayed using ESPript 3^39^.

### Crystallization of OPRT from *M. tuberculosis*

Initial crystallization screens were performed at 4 °C by the sitting-drop vapour-diffusion method using commercially available screens (Qiagen, Hampton Research, Molecular Dimensions) and using an Oryx4 Protein Crystallization Robot (Douglas Instruments Ltd.). For initial crystallization screens, 0.5 µl of protein solution was mixed with 0.5 µl of reservoir solution, and the final drop was equilibrated against 50 µl of reservoir solution. The inorganic phosphate-bound and PRPP-bound enzyme (enzyme concentration of 5.4 mg/ml) crystallized in condition 37 of the Crystal Screens II (0.1 M HEPES pH 7.5, 10% (w/v) PEG8000, 8% (v/v) Ethylene glycol, 1 mM PRPP) and condition 93 of Classic suite I (0.1 M HEPES pH 7.5, 10% (w/v) PEG6000, 5% (v/v) MPD, 1 mM PRPP), respectively. Crystals of MtOPRT in complex with FDC (enzyme concentration of 3.4 mg/ml) where obtained following optimizations of condition 18 of Crystal screen II in a final reservoir solution containing 0.01 M Iron(III) chloride hexahydrate, 0.1 M sodium citrate tribasic dihydrate pH 6.1, 10% (v/v) and Jeffamine M-600. Crystals were fished and cryoprotected with 25% (w/v) glycerol for data collection and storage in liquid nitrogen.

### X-Ray Data Collection, Structure Determination and Refinement

MtOPRT crystals diffraction data were collected at the ESRF in Grenoble (France) on beamline ID29 for the MtOPRT-inorganic phosphate and -FDC complexes, and on beamline ID23–1 for the MtOPRT-PRPP complex. Diffraction data were processed using XDS^40^ and scaled using SCALA of the CCP4 program suite^41^. The structure of MtOPRT- FDC complex was solved by molecular replacement using the program PHASER^42^ of the PHENIX program suite^28^ and using the structure of phosphoribosyltransferase from Corynebacterium diphtheriae as the search model (PDB code: 2P1Z). Automatic model building was performed using ARP-wARP CLASSIC^43^ of the CCP4 suite^41^, and Coot^44^ was used for manual model building. Refinement was carried out using REFMAC5^45^ and PHENIX^28^.

The MtOPRT-FDC crystal belonged to the triclinic space group (P1) with unit cell parameters a = 52.4 Å, b = 60.3 Å, c = 65.2 Å, α = 85.6° β = 89.9° γ = 80.0° and diffracted to a resolution of 2.40 Å. The asymmetric unit contained four protein molecules organised as two independent catalytic dimers. All the four polypeptide chains present in the asymmetric unit exhibited a well-defined electron density; chains A and B will be used as reference throughout the manuscript.

The structures for the enzyme bound to inorganic phosphate and PRPP were determined by molecular replacement using PHASER^42^ and chain A from the refined, ligand-free MtOPRT structure as the search model. The crystals of the MtOPRT-PRPP complex was assigned to space group P2_1_ with unit cell parameters a = 56.2 Å, b = 58.6 Å, c = 57.2 Å, α = 90° β = 116.9° γ = 90.0°, and diffracted to a resolution of 2.25 Å.

The MtOPRT-Pi complex crystallized in space group P2_1_ with unit cell parameters a = 57.3 Å, b = 56.3 Å, c = 59.5 Å, α = 90.0° β = 115.1° γ = 90.0° and diffracted to a resolution of 1.90 Å, with the asymmetric unit containing one functional dimer.

The stereochemistry of the structures has been assessed with the program PROCHECK^46^. Data collection and refinement statistics are summarized in Table 3. Interaction diagrams were prepared using the program LIGPLOT^47^. All the structure-related figures were produced using PyMOL^48^.

**Table 3:** Data collection and refinement statistics^a^.

	MtOPRT-Pi	MtOPRT-PRPP	MtOPRT-FDC
**Data collection**			
Diffraction source	Beamline ID29, ESRF	Beamline ID23-1, ESRF	Beamline ID29, ESRF
Wavelength (Å)	0.9	1.899	0.9762
Temperature (K)	100	100	100
Detector	Pilatus 6M	Q315R	Pilatus 6M
Crystal-to-detector distance (mm)	355.93	181.12	455.14
Rotation range per image (°)	0.05	0.15	0.1
Space group	P 2_1_	P 2_1_	P 1
**Unit cell**			
a, b, c (Å)	57.3, 56.3, 59.5	56.2, 58.6, 57.2	52.4, 60.3, 65.2
α, β, γ (°)	90.0, 115.1, 90.0	90.0, 116.86, 90.0	85.6, 89.9, 80.0
Resolution range (Å)	53.85–1.90 (1.97–1.90)	51.05–2.25 (2.33–2.25)	42.17–2.40 (2.49–2.40)
Total reflections	86801 (12158)	73436 (10002)	108116 (10667)
Unique reflections	26976 (2583)	15770 (1559)	29148 (2860)
Multiplicity	3.2 (3.2)	4.7 (4.4)	3.7 (3.7)
Completeness (%)	99.00 (95.53)	99.19 (99.49)	95.3 (94.4)
Mean I/sigma(I)	13.61 (4.78)	19.66 (7.08)	8.74 (2.78)
Overall B factor from Wilson plot (Å)	19.4	27.6	31.6
R-merge	0.056 (0.20)	0.045 (0.13)	0.11 (0.43)
Molecule per asymmetric Unit	2	2	4
**Refinement statistics**			
Rwork/Rfree	0.15/0.19 (0.20/0.25)	0.20/0.24 (0.25/0.29)	0.19/0.25 (0.255/0.37)
Protein	2546	2456	5285
Phosphate	5		
PRPP		44	
FDC			54
Water	208	86	169
Total	2759	2586	5337
R.m.s deviation bonds (Å)/Angles (°)	0.018/1.78	0.013/1.82	0.008/1.18
Clashscore	3.92	6.41	7.65
**Ramachandran plot**			
Favored regions (%)	98.5	97.8	98.5
Additional allowed (%)	0.9	1.9	0
Outliers (%)	0.6	0.3	0.0
PDB ID code	5HKL	5HKF	5HKI

^a^Values in parenthesis are for the highest-resolution shell.

## Electronic supplementary material

Supplemetary materials

Molecular dynamics simulation movie of MtOPRTase

## References

[1] A D Silva PEA, Palomino JC (2011). Molecular basis and mechanisms of drug resistance in Mycobacterium tuberculosis: classical and new drugs. J. Antimicrob. Chemother..

[2] Gandhi NR (2010). Multidrug-resistant and extensively drug-resistant tuberculosis: a threat to global control of tuberculosis. Lancet Lond. Engl..

[3] World Health Organization. Global Tuberculosis Report 2015 (WHO, 2015).

[4] Zhang Y (2005). The Magic Bullets and Tuberculosis Drug Targets. Annu. Rev. Pharmacol. Toxicol..

[5] Mitchison DA (2004). The search for new sterilizing anti-tuberculosis drugs. Front. Biosci. J. Virtual Libr..

[6] Duncan K, Barry CE (2004). Prospects for new antitubercular drugs. Curr. Opin. Microbiol..

[7] Smith CV, Sharma V, Sacchettini JC (2004). TB drug discovery: addressing issues of persistence and resistance. Tuberc. Edinb. Scotl..

[8] Kremer LS, Besra GS (2002). Current status and future development of antitubercular chemotherapy. Expert Opin. Investig. Drugs.

[9] Warner, D. F., Evans, J. C. & Mizrahi, V. Nucleotide Metabolism and DNA Replication. *Microbiol. Spectr*. **2** (2014). 10.1128/microbiolspec.MGM2-0001-2013 26104350

[10] Turnbough CL, Switzer RL (2008). Regulation of Pyrimidine Biosynthetic Gene Expression in Bacteria: Repression without Repressors. Microbiol. Mol. Biol. Rev. MMBR.

[11] Breda A, Rosado LA, Lorenzini DM, Basso LA, Santos DS (2012). Molecular, kinetic and thermodynamic characterization of Mycobacterium tuberculosis orotate phosphoribosyltransferase. Mol. Biosyst..

[12] Breda A (2012). Pyrimidin-2(1H)-ones based inhibitors of Mycobacterium tuberculosis orotate phosphoribosyltransferase. Eur. J. Med. Chem..

[13] Cleland WW (1967). Enzyme Kinetics. Annu. Rev. Biochem..

[14] Rebholz, K. L. & Northrop, D. B. In (ed. Enzymology, B.-M. in) 249, 211–240 (Academic Press, 1995). 10.1016/0076-6879(95)49037-x 7791613

[15] Northrop DB, Rebholz KL (1997). Kinetics of Enzymes with Iso-Mechanisms: Solvent Isotope Effects. Arch. Biochem. Biophys..

[16] Krungkrai SR (2004). Human malaria parasite orotate phosphoribosyltransferase: functional expression, characterization of kinetic reaction mechanism and inhibition profile. Mol. Biochem. Parasitol..

[17] Javaid ZZ, el Kouni MH, Iltzsch MH (1999). Pyrimidine nucleobase ligands of orotate phosphoribosyltransferase from Toxoplasma gondii. Biochem. Pharmacol..

[18] Wang GP, Hansen MR, Grubmeyer C (2012). Loop residues and catalysis in OMP synthase. Biochemistry.

[19] González-Segura L, Witte JF, McClard RW, Hurley TD (2007). Ternary complex formation and induced asymmetry in orotate phosphoribosyltransferase. Biochemistry.

[20] Kumar S, Krishnamoorthy K, Mudeppa DG, Rathod PK (2015). Structure of Plasmodium falciparum orotate phosphoribosyltransferase with autologous inhibitory protein-protein interactions. Acta Crystallogr. Sect. F Struct. Biol. Commun.

[21] Henriksen A, Aghajari N, Jensen KF, Gajhede M (1996). A flexible loop at the dimer interface is a part of the active site of the adjacent monomer of Escherichia coli orotate phosphoribosyltransferase. Biochemistry.

[22] Scapin G, Ozturk DH, Grubmeyer C, Sacchettini JC (1995). The crystal structure of the orotate phosphoribosyltransferase complexed with orotate and alpha-D-5-phosphoribosyl-1-pyrophosphate. Biochemistry.

[23] Liu CP (2010). Structure of orotate phosphoribosyltransferase from the caries pathogen Streptococcus mutans. Acta Crystallograph. Sect. F Struct. Biol. Cryst. Commun..

[24] Wang GP, Cahill SM, Liu X, Girvin ME, Grubmeyer C (1999). Motional dynamics of the catalytic loop in OMP synthase. Biochemistry.

[25] Grubmeyer C, Hansen MR, Fedorov AA, Almo SC (2012). Structure of Salmonella typhimurium OMP Synthase in a Complete Substrate Complex. Biochemistry.

[26] Silva, A. M. N., Kong, X., Parkin, M. C., Cammack, R. & Hider, R. C. Iron(III) citrate speciation in aqueous solution. *Dalton Trans. Camb. Engl. 2003* 8616–8625, 10.1039/b910970f (2009). 10.1039/b910970f 19809738

[27] Pierre JL, Gautier-Luneau I (2000). Iron and citric acid: a fuzzy chemistry of ubiquitous biological relevance. Biometals Int. J. Role Met. Ions Biol. Biochem. Med..

[28] Adams PD (2010). PHENIX: a comprehensive Python-based system for macromolecular structure solution. Acta Crystallogr. D Biol. Crystallogr..

[29] Scapin G, Grubmeyer C, Sacchettini JC (1994). Crystal structure of orotate phosphoribosyltransferase. Biochemistry.

[30] Crichton, R. In *Inorganic Biochemistry of Iron Metabolism* 17–48 (John Wiley & Sons, Ltd, 2001).

[31] Thauer RK (1988). Citric-acid cycle, 50 years on. Modifications and an alternative pathway in anaerobic bacteria. Eur. J. Biochem. FEBS.

[32] Fukushima T (2012). Bacillus cereus iron uptake protein fishes out an unstable ferric citrate trimer. Proc. Natl. Acad. Sci. USA.

[33] Vukosav P, Mlakar M, Tomišić V (2012). Revision of iron(III)-citrate speciation in aqueous solution. Voltammetric and spectrophotometric studies. Anal. Chim. Acta.

[34] Franz KJ (2012). Application of inorganic chemistry for non-cancer therapeutics. Dalton Trans..

[35] Ortega-Carrasco E, Lledós A, Maréchal J-D (2014). Assessing protein–ligand docking for the binding of organometallic compounds to proteins. J. Comput. Chem..

[36] Bradford MM (1976). A rapid and sensitive method for the quantitation of microgram quantities of protein utilizing the principle of protein-dye binding. Anal. Biochem..

[37] Notredame C, Higgins DG, Heringa J (2000). T-Coffee: A novel method for fast and accurate multiple sequence alignment. J. Mol. Biol..

[38] O’Sullivan O, Suhre K, Abergel C, Higgins DG, Notredame C (2004). 3DCoffee: combining protein sequences and structures within multiple sequence alignments. J. Mol. Biol..

[39] Robert X, Gouet P (2014). Deciphering key features in protein structures with the new ENDscript server. Nucleic Acids Res..

[40] Kabsch W (2010). XDS. Acta Crystallogr. D Biol. Crystallogr..

[41] Collaborative Computational Project. Number 4. The CCP4 suite: programs for protein crystallography. *Acta Crystallogr. D Biol. Crystallogr*. **50**, 760–763 (1994). 10.1107/S0907444994003112 15299374

[42] McCoy AJ (2007). Phaser crystallographic software. J. Appl. Crystallogr..

[43] Langer G, Cohen SX, Lamzin VS, Perrakis A (2008). Automated macromolecular model building for X-ray crystallography using ARP/wARP version 7. Nat. Protoc..

[44] Emsley P, Lohkamp B, Scott WG, Cowtan K (2010). Features and development of Coot. Acta Crystallogr. D Biol. Crystallogr..

[45] Murshudov GN (2011). REFMAC5 for the refinement of macromolecular crystal structures. Acta Crystallogr. D Biol. Crystallogr..

[46] Vaguine AA, Richelle J, Wodak SJ (1999). SFCHECK: a unified set of procedures for evaluating the quality of macromolecular structure-factor data and their agreement with the atomic model. Acta Crystallogr. D Biol. Crystallogr..

[47] Wallace AC, Laskowski RA, Thornton JM (1995). LIGPLOT: a program to generate schematic diagrams of protein-ligand interactions. Protein Eng..

[48] DeLano, W. L. & Lam, J. W. PyMOL: A communications tool for computational models. *Abstr Pap Am Chem Soc* **230**, U1371–U1372 (2005).

