# Supplementary material for: Structural investigations on orotate phosphoribosyltransferase from Mycobacterium tuberculosis, a key enzyme of the de novo pyrimidine biosynthesis
Source: Sci Rep. 2017 Apr 26;7:1180. doi: 10.1038/s41598-017-01057-z (PMC5430920; doi:10.1038/s41598-017-01057-z)
Supplement: Supplementary file 1 — Supplemetary materials [file 41598_2017_1057_MOESM1_ESM.doc]

**Structural investigations on orotate phosphoribosyltransferase from *Mycobacterium tuberculosis,* a key enzyme of the *de novo* pyrimidine biosynthesis**

Stefano Donini^1,+^, Davide M Ferraris^1,+^, Riccardo Miggiano^1^, Alberto Massarotti^1^, Menico Rizzi^1^,*

^1^Department of Pharmaceutical Sciences, Università del Piemonte Orientale “A. Avogadro”, Largo Donegani 2, 28100 Novara, Italy

^+^These authors contributed equally to the work

*[menico.rizzi@uniupo.it](mailto:menico.rizzi@uniupo.it)

**Supplementary Materials**

**Molecular dynamics (MD)**

The crystallographic structure of ligand-free MtOPRTase (PDB ID: 5HKL, chain B) was used for the initial coordinates of the MD simulations. MD simulation was performed using GROMACS 5.1 software^1^ with the GROMOS 54a7 force field^2^ implemented in Intel Xeon Octa Core processor with Linux environment. In our model, basic residues are protonated and acidic residues are unprotonated. Systems were neutralized and solvated in a periodic octahedric box containing a simple point charge (SPC) water model. A simulation was carried out at a constant temperature of 300 K. Before MD simulation, the internal constraints were relaxed by energy minimization, followed by equilibration (100 ps at constant temperature and 100 ps at constant pressure) under position restraints of the carbon backbone atoms. During the MD run, covalent bonds in the protein were constrained using the LINCS algorithm^3^. The SETTLE algorithm was used to constrain the geometry of water molecules^4^. Berendsen’s coupling algorithm was used to maintain the simulation under constant pressure and temperature^5^. Van der Waals forces were treated using a cut-off of 1.0 nm. Long range electrostatic forces (r. 1.0 nm) were treated using particle mesh implemented in the Verlet method^6^. Through the production runs, the trajectory data were saved every 1 ps, and the total duration of the simulation was 200 ns. Root-mean-square deviations (RMSD) were calculated taking the energy-minimized structure as a reference (Supplementary Figure S2). Principal components analysis (PCA) of the protein motion was determined from the diagonalization of the covariance matrix of the interatomic fluctuation (Supplementary Figure S3)^7^. Average conformations were calculated from the variance-covariance matrix of all protein atoms during the equilibrium time of the run. Tools from the GROMACS package were used for the analysis of the data.


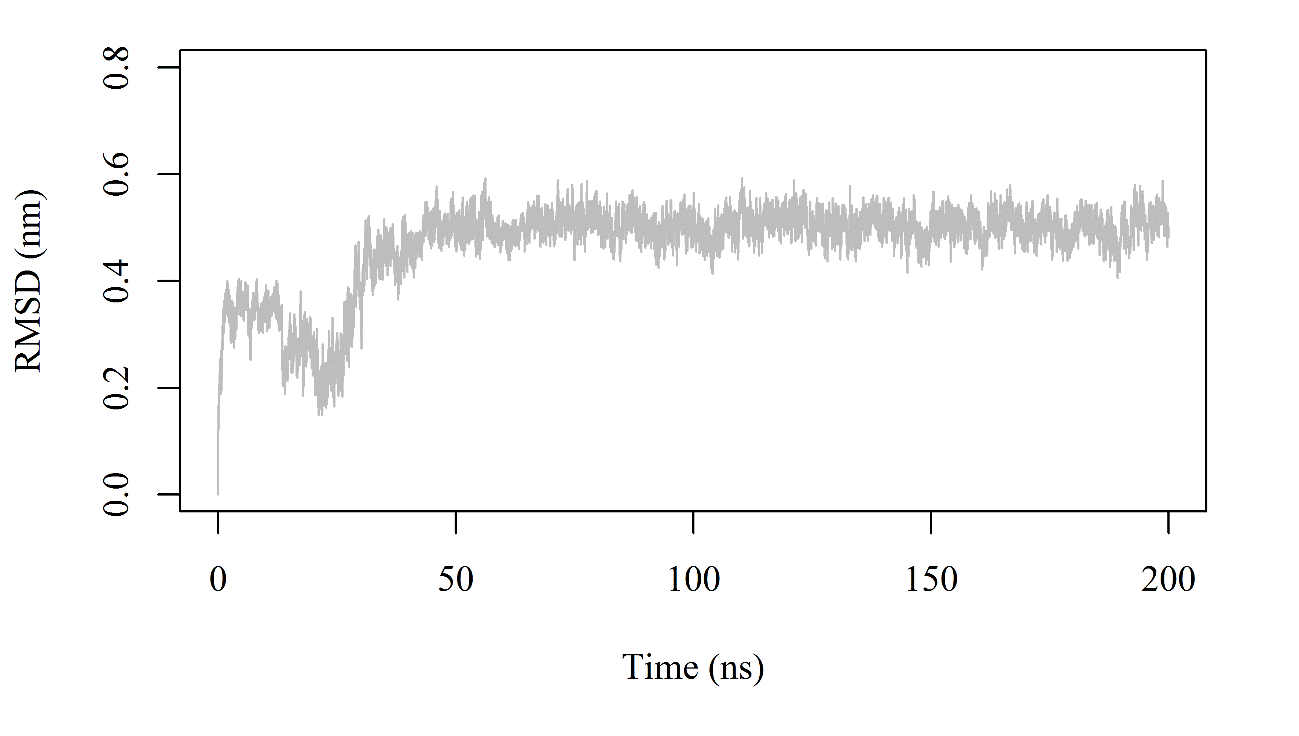


**Figure S1.** Root mean square deviation (RMSD) from the crystallographic structures of the Cα atoms as a function of simulation time for MtOPRT without ligand. Simulation was performed at 300 K.


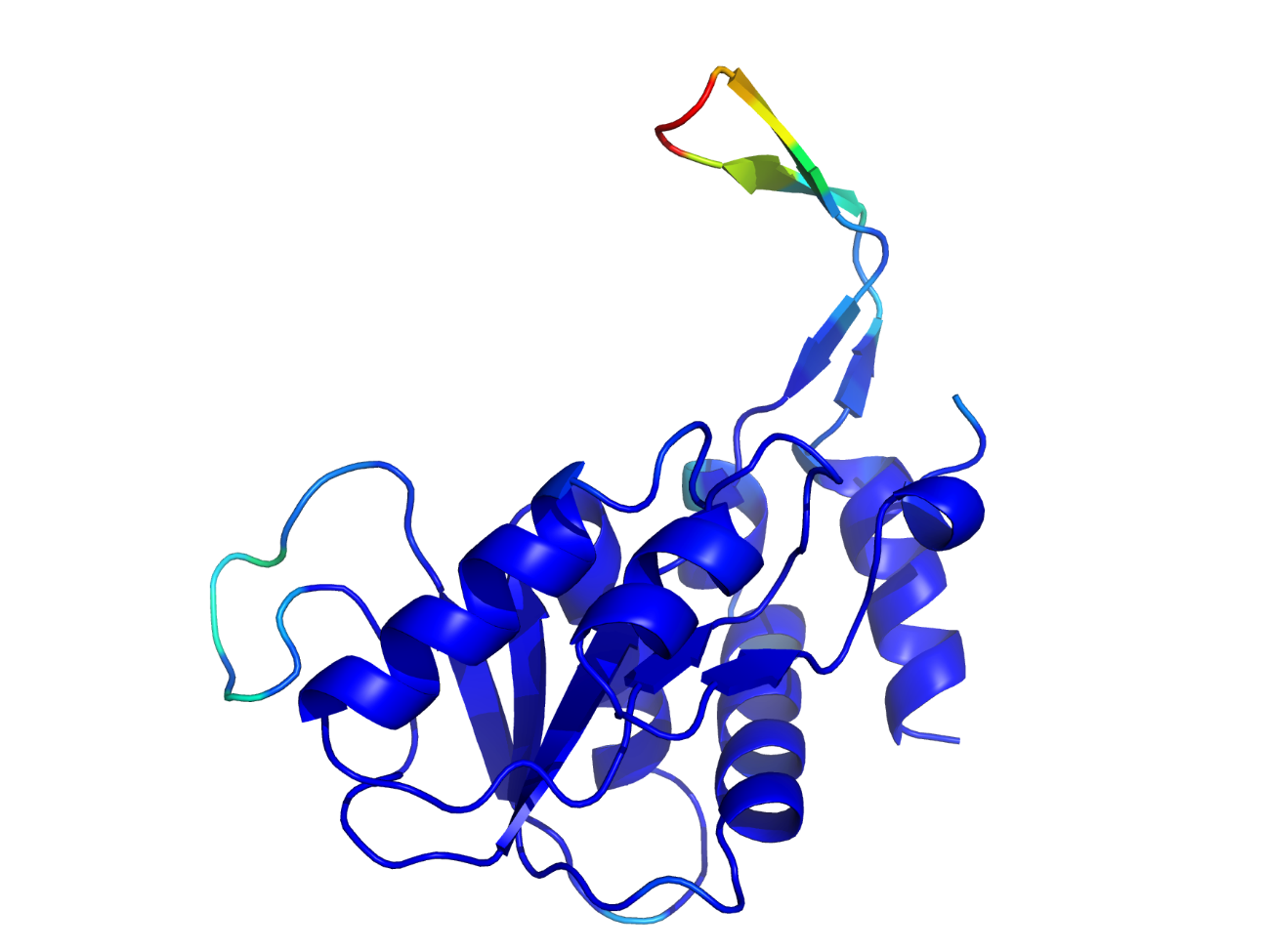


**Figure S2.** PCA analysis of MtOPRT MD simulation. PCA analysis was performed to analyse the dynamics of residues during the MD simulation. A colour scale is used to indicate the stable (blue) and unstable (red) portions of the protein structure.


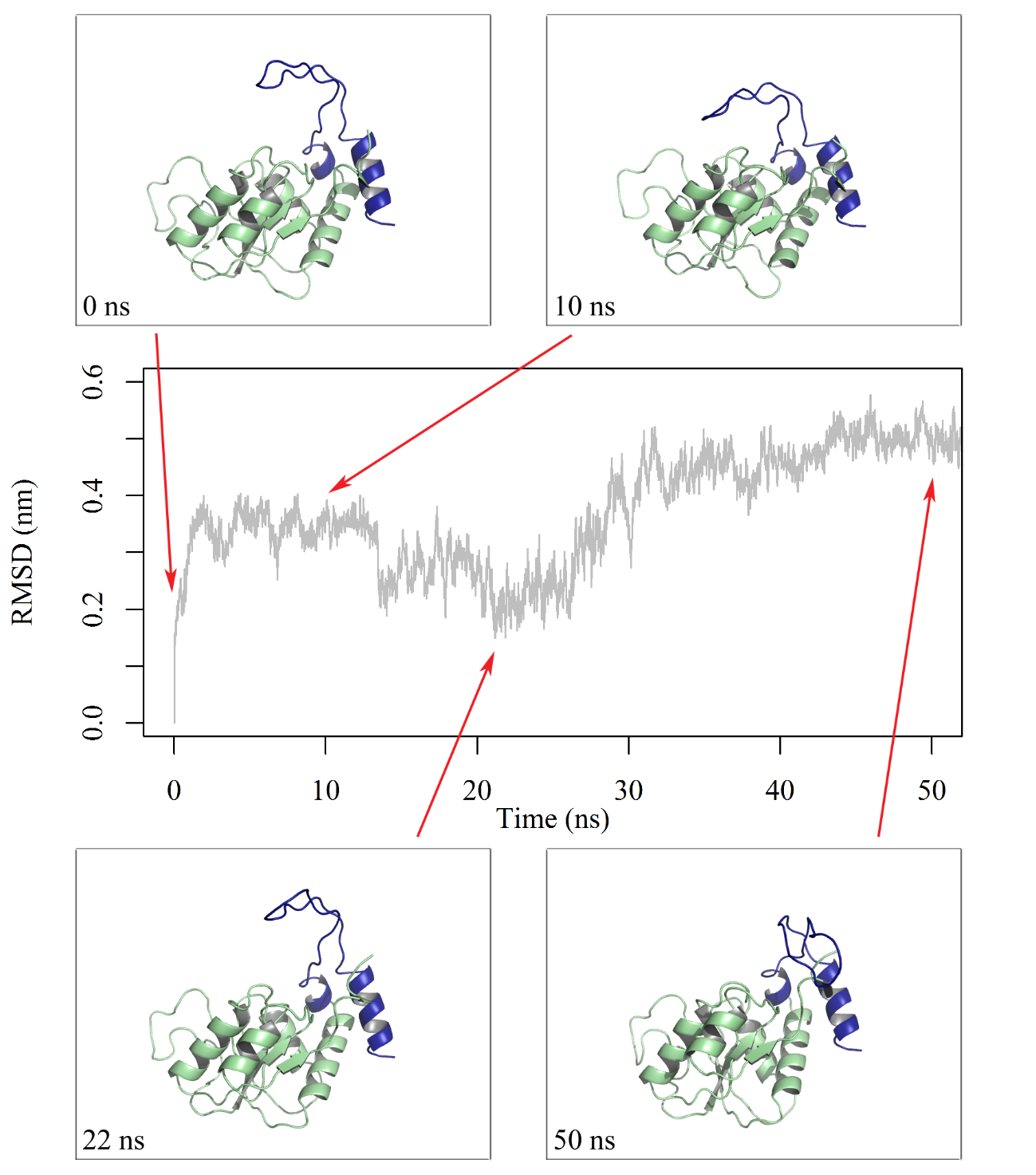


**Figure S3.** Detail of root mean square deviation (RMSD) from the crystallographic structures of the Cα atoms as a function of simulation time for MtOPRT without ligand. Most representative conformations are depicted, central ɑ/β core and hood domain are in palegreen and blue respectively. Movie of the MD simulation is available as Supporting Materials.

**Video legend:**

Molecular dynamics simulation movie of MtOPRTase. Shown is a cartoon representation of the 200-nanosecond trajectory, monomer A is coloured in green; hood domain is coloured in blue.

**References**

1. Abraham, M. J. *et al.* GROMACS: High performance molecular simulations through multi-level parallelism from laptops to supercomputers. *SoftwareX* **1–2,** 19–25 (2015).

2. Schmid, N. *et al.* Definition and testing of the GROMOS force-field versions 54A7 and 54B7. *Eur. Biophys. J. EBJ* **40,** 843–856 (2011).

3. Hess, B., Bekker, H., Berendsen, H. J. C. & Fraaije, J. G. E. M. LINCS: A linear constraint solver for molecular simulations. *J. Comput. Chem.* **18,** 1463–1472 (1997).

4. Miyamoto, S. & Kollman, P. A. Settle: An analytical version of the SHAKE and RATTLE algorithm for rigid water models. *J. Comput. Chem.* **13,** 952–962 (1992).

5. Berendsen, H. J. C., Postma, J. P. M., Gunsteren, W. F. van, DiNola, A. & Haak, J. R. Molecular dynamics with coupling to an external bath. *J. Chem. Phys.* **81,** 3684–3690 (1984).

6. Verlet, L. Computer ‘Experiments’ on Classical Fluids. I. Thermodynamical Properties of Lennard-Jones Molecules. *Phys. Rev.* **159,** 98–103 (1967).

7. Amadei, A., Linssen, A. B. M. & Berendsen, H. J. C. Essential dynamics of proteins. *Proteins Struct. Funct. Bioinforma.* **17,** 412–425 (1993).
